# Three complete mitochondrial genomes of the genus *Mudaria* (Lepidoptera, Noctuidae) with phylogenetic significance

**DOI:** 10.3897/zookeys.1296.206586

**Published:** 2026-11-06

**Authors:** Yangjie Wang, Zhiwei Dong, Zonghan Deng, Jinwu He, Yuanrong Li, Lijie Zhang, Pisit Poolprasert, Markku Pellinen, Huilin Han, Xueyan Li

**Affiliations:** 1 Key Laboratory of Genetic Evolution & Animal Models, Kunming Institute of Zoology, Chinese Academy of Sciences, Kunming 650223, China Key Laboratory of Genetic Evolution & Animal Models, Kunming Institute of Zoology, Chinese Academy of Sciences Kunming China; 2 University of Chinese Academy of Sciences, Beijing 101408, China University of Chinese Academy of Sciences Beijing China; 3 Kunming Customs Technical Center, Kunming 650228, China Kunming Customs Technical Center Kunming China; 4 Comprehensive Technical Center, Mengla Customs, Mengla 666301, China Comprehensive Technical Center, Mengla Customs Mengla China; 5 Science and Technology Research Center of China Customs, Beijing 100026, China Science and Technology Research Center of China Customs Beijing China; 6 Department of Entomology, Faculty of Agriculture, Kasetsart University, Bangkok 10900, Thailand Department of Entomology, Faculty of Agriculture, Kasetsart University Bangkok Thailand; 7 175 M1 Muban Phichai, Mueang Lampang, 52000 Thailand Unafilliated Mueang Lampang Thailand; 8 Northeast Asia Biodiversity Research Center, Northeast Forestry University, Harbin 150040, China Northeast Asia Biodiversity Research Center, Northeast Forestry University Harbin China; 9 Yunnan Key Laboratory of Biodiversity Information, Kunming Institute of Zoology, Chinese Academy of Sciences, Kunming 650223, China Yunnan Key Laboratory of Biodiversity Information, Kunming Institute of Zoology, Chinese Academy of Sciences Kunming China

**Keywords:** Durian-fruit borers, mitogenome, *
Mudaria
*, pest management, phylogenetic relationships

## Abstract

*Mudaria* Moore, 1893 (Lepidoptera, Noctuidae), the common durian-fruit borers, are economically devastating pests of tropical crops (such as *Durio zibethinus*, *Ceiba pentandra*, *Bombax* spp.), yet their phylogenetic position has long been controversial due to morphological ambiguities. In this study, we sequenced and annotated three complete mitochondrial genomes (mitogenomes) of this genus: *Mudaria szalkayjozsefi* (15,325 bp), *Mudaria bogdanpopescui* (15,391 bp), and *Mudaria stahlgretschae* (15,396 bp). All these mitogenomes exhibit the conserved ancestral lepidopteran gene order of 37 genes and display a profound A+T compositional bias (>80%). Comparative mitogenomic analyses revealed highly conserved relative synonymous codon usage and typical invertebrate mitochondrial features, alongside notable interspecific variation in the start codon of the *atp8* gene (165/159/165 bp). To clarify the phylogenetic relationships of *Mudaria* among Noctuidae, a robust macro-scale maximum-likelihood phylogenetic framework was reconstructed using a concatenated super-matrix (13 protein-coding genes and two rRNA genes) from 144 species, alongside an expanded fine-scale *cox1* tree of 249 species. Crucially, our super-matrix unambiguously placed the *Mudaria* clade as an independent basal lineage of Noctuidae, positioned as the sister group to the subfamily Acontiinae. Concurrently, the fine-scale *cox1* tree pinpointed its precise evolutionary neighborhood, resolving *Mudaria* as the immediate sister group to the Acontiine lineage comprising *Parerastria castaneata* and *Lacistophanes hackeri*. Although a formal subfamilial reassignment remains tentative pending comprehensive morphological validation, these newly curated genomic and barcode references provide critical high-precision anchors for international quarantine interception and the refinement of integrated pest management strategies against these destructive cryptic pests.

## Introduction

Durian (*Durio zibethinus* L.), widely recognized as the “king of fruits”, is a tropical fruit crop of substantial economic importance in Southeast Asia. The global durian market has experienced significant growth, with increasing international demand driving the expansion of cultivated areas and export volumes (Jittamai et al. 2024). This high-value commodity, however, faces persistent threats from a range of phytosanitary issues, with insect pests posing a major constraint to sustainable production (Pinsorn and Sirikantaramas 2025). Among the most damaging pests is the durian-fruit borer, *Mudaria luteileprosa* Holloway, 1989 (Lepidoptera, Noctuidae) (Beckmann et al. 2009). Historically, investigations into the seasonal occurrence and population dynamics of durian-fruit borers in Southeast Asia primarily identified the infesting agent as *Mudaria magniplaga* Walker, 1858 (Lee 1996). However, subsequent taxonomic reassessments indicated that many historic records of economic damage within commercial durian orchards actually involved *M. luteileprosa* or other cryptic congeners. More recently, *Mudaria stahlgretschae* Ronkay, Ronkay, Pekarsky & Landry, 2023 has also emerged as a primary threat in the region, causing severe cryptic damage by boring directly into the fruit seeds (Chamsuk et al. 2026). Infestations by these species cause direct damage to the fruit pulp and seeds, leading to significant yield losses and post-harvest rejection. Economically, these pests incur significant losses through export rejections of durian. These disruptions not only compromise market access but also necessitate stricter phytosanitary compliance for exporters (Jittamai et al. 2024). The accurate identification, classification, and understanding of the biology of *Mudaria* species are fundamental prerequisites for the development of effective quarantine and integrated pest management strategies.

The genus *Mudaria* Moore, 1893 comprises approximately 30 species. Adults of the genus are easily characterized by the diagnostic structures of the genitalia of both sexes, the typical structure of the abdominal sclerites (Ronkay et al. 2022), but on the other hand, the ground plan of the male genitalia is surprisingly similar throughout the entire genus (Ronkay et al. 2023). This diagnostic challenge is further heightened by the co-occurrence of other congeners, such as *Mudaria cornifrons* Moore, 1893 and *Mudaria variabilis* Roepke, 1916, which are morphologically nearly indistinguishable from the primary durian-boring pests. However, these sibling species are typically associated with alternative Malvaceae hosts or tend to occur outside the typical durian cropping season (Chamsuk et al. 2026). The larvae of this genus are specialized feeders on plants of the families Malvaceae*sensu lato*, including Bombacaceae and Sterculiaceae, with recorded hosts including durian (*D. zibethinus*), kapok (*Ceiba pentandra* (L.) Gaertn.), silk-cotton tree (*Bombax* L. spp.), and *Pterospermum* Schreb. species (Roepke 1916; Holloway 1989; Robinson et al. 2001). This endophytic feeding habit, in which larvae bore into fruits or pods, renders the genus of particular economic significance, especially in the context of international trade of durian and other tropical fruits. The subfamilial affiliation of *Mudaria* has been a subject of long-standing taxonomic debate, with the genus being frequently reassigned across disparate lineages of Noctuidae due to ambiguous or conflicting morphological traits. Holloway (1989) classified the genus within Hadeninae, tentatively associating it with the genus *Chasmina* Walker, 1856 based on general morphological similarities. Kononenko (2013) later shifted it to Condicinae, reflecting different interpretations of its ambiguous morphological traits. Highlighting this persistent systematic uncertainty, Pellinen et al. (2018) prudently treated *Mudaria* as Noctuinae incertae sedis due to the lack of definitive synapomorphies linking it to any specific lineage. Most recently, Ronkay et al. (2022, 2023) proposed its placement within the subfamily Psaphidinae, relying primarily on the structural ground plan of the male genitalia and the typical configuration of the abdominal sclerites. This taxonomic ambiguity hinders our understanding of the group’s evolutionary history and impedes accurate species identification and phylogenetic inference. Although molecular datasets are increasingly integrated with traditional morphological diagnoses to resolve systematic relationships and cryptic species boundaries within Lepidoptera, only nine species of *Mudaria* have *cox1* barcode sequences available in the National Center for Biotechnology Information (NCBI).

Mitochondrial genomes (mitogenomes) have emerged as powerful molecular markers for insect phylogenetic studies. Characterized by their conserved gene content, rapid evolutionary rate, and maternal inheritance, mitogenomes provide a wealth of phylogenetic information that is particularly effective for resolving relationships at various taxonomic levels (Niu et al. 2024). The complete mitogenome, typically encompassing 13 protein-coding genes (PCGs), 22 transfer RNA (tRNA) genes, two ribosomal RNA (rRNA) genes, and a large non-coding control region, offers a substantial number of informative characters for phylogenetic reconstruction. Recent studies have demonstrated the utility of mitogenomic data in clarifying the phylogenetic positions of insect groups with contested classifications and providing robust molecular diagnostic tools to separate cryptic or morphologically similar species (Shah et al. 2022; Riyaz et al. 2023).

In this study, we present the first complete mitochondrial genome sequences for three species of *Mudaria*: *M. szalkayjozsefi* Ronkay, Ronkay & Pekarsky, 2023, *M. bogdanpopescui* Ronkay, Ronkay, Pekarsky & Landry, 2023, and *M. stahlgretschae*. We characterize and compare their sequence structural features with other moths, including nucleotide composition, gene arrangement, and codon usage. Phylogenetic analyses were performed to infer the precise phylogenetic position of *Mudaria* within Noctuidae based on comprehensive mitogenomic data. Our results provide key insights into the longstanding subfamilial affiliation of this economically important genus and provide vital data resources for future taxonomic, ecological, and applied research, ultimately contributing to improved pest management, accurate cryptic species diagnosis, and quarantine strategies.

## Materials and methods

### Sample collection and identification

**Figure 1.**
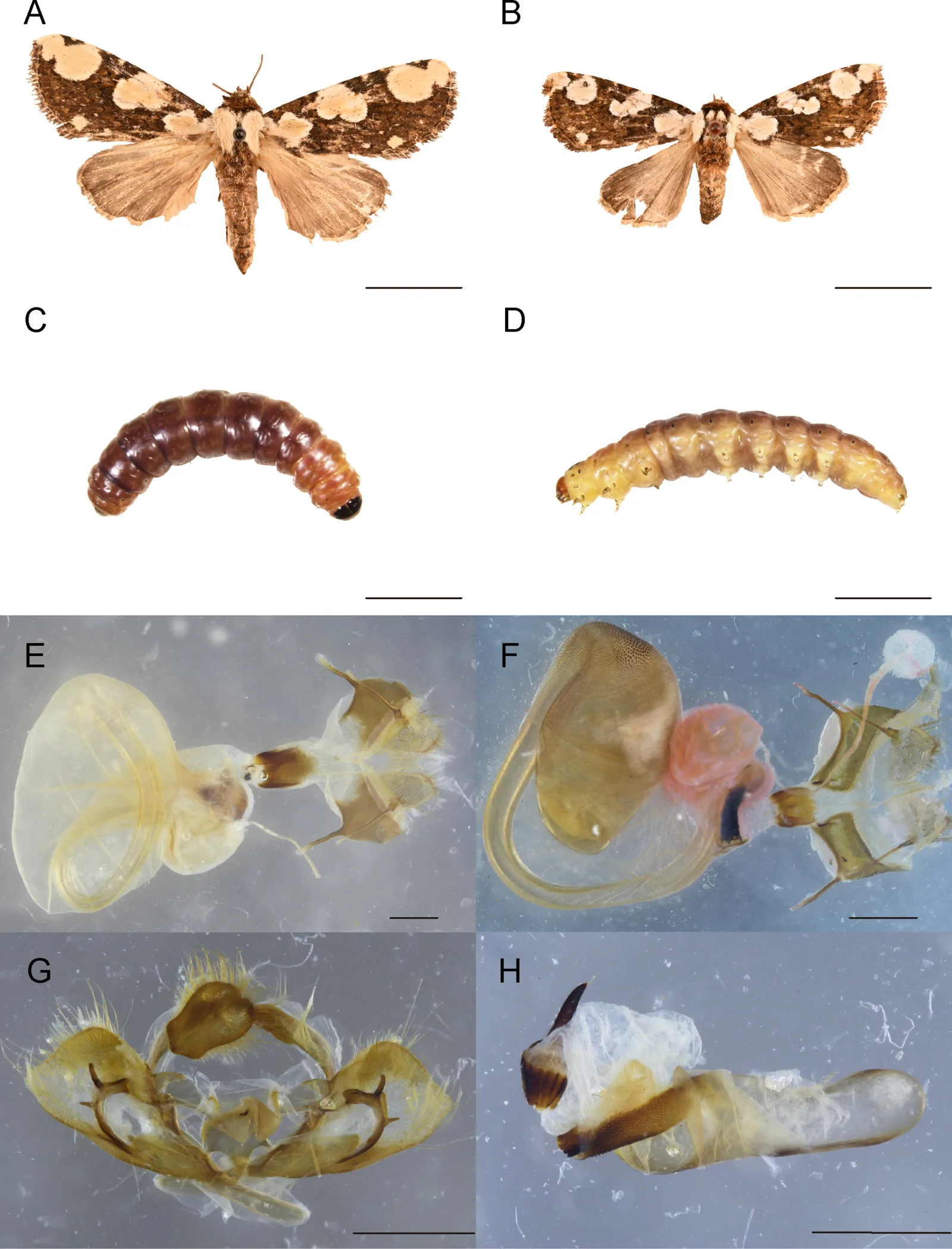
Morphological features and genitalia structures of *Mudaria* species in this study. **A**. *M. bogdanpopescui*, adult; **B**. *M. szalkayjozsefi*, adult; **C, D**. *M. stahlgretschae*, larva, dorsal view (**C**), lateral and ventral view (**D**); **E**. Female genitalia of *M. bogdanpopescui*; **F–H**. Genitalia of *M. szalkayjozsefi*, female genitalia (**F**), male genitalia valva (**G**) and aedeagus (**H**). Scale bars: 10 mm (**A–D**); 1 mm (**E–H**).

Adult specimens of *M. szalkayjozsefi* (two females and three males) and *M. bogdanpopescui* (two females) were collected using light traps in Gelang Patah, Johor, Malaysia, in June 2025. Taxonomic identification of these two species was performed based on diagnostic characters of external adult morphology and thorough examinations of both male and female genitalia structures (Fig. 1) (Ronkay et al. 2023). Some larvae of *M. stahlgretschae* were intercepted in Mohan Port from imported durian fruits in August 2023. Two of them were submitted to the Plant Quarantine Laboratory of the Mengla Customs Comprehensive Technology Center for laboratory rearing, and one of the larvae underwent pupation and subsequently emerged as a female adult moth. One of them was submitted to Kunming Customs Technical Center for DNA barcoding data. Two female larvae of *M. stahlgretschae* were collected from imported durian fruits bought in the Jinma Zhengchang Fruit Wholesale Market in Kunming, Yunnan Province, China, in August 2024 (Fig. 1), and one of the larvae was used for next-generation sequencing. The identification of *M. stahlgretschae* was confirmed via sequence alignment based on DNA barcoding (GenBank accession PZ250739) (Chamsuk et al. 2026).

### DNA extraction and sequencing

To preserve the morphological diagnostic features of the adult voucher specimens, three unilateral legs were carefully dissected from each individual of *M. bogdanpopescui*, *M. szalkayjozsefi*, and *M. stahlgretschae*. For the larval specimens of *M. stahlgretschae*, longitudinal muscle tissues were sampled. All dissected tissues were preserved in absolute ethanol. DNA of *M. stahlgretschae* for DNA barcoding was extracted from larval abdomen tissue using TGuide Smart Universal Genomic DNA Extraction Kit (TIANGEN); amplification of the *cox1* animal DNA barcode sequence was performed using the universal primers LCO1490 (GGTCAACAAATCATAAAGATATTGG) and HCO2198 (TAAACTTCAGGGTGACCAAAAAATCA), and the amplification product was sequenced at Sangon Biotech (Shanghai). DNA extraction, shotgun library construction, and next-generation sequencing of *M. bogdanpopescui* (adult), *M. szalkayjozsefi* (adult), and *M. stahlgretschae* (larvae) were entirely performed by BGI Genomics (Shenzhen, China). The libraries were sequenced on the DNBSEQ-T7 high-throughput platform with a paired-end 150 bp (PE150) strategy, generating approximately 1.00 Gb of raw data per specimen.

### Mitogenome assembly, annotation, and visualization

The complete mitogenomes were *de novo* assembled using NOVOPlasty v. 4.3.1 (Dierckxsens et al. 2016). The assemblies were initially annotated using the MITOS2 pipeline (Bernt et al. 2013) based on the refseq63m reference database, with the invertebrate mitochondrial genetic code (Code 5). The 13 protein-coding genes (PCGs) and two rRNAs were also checked manually by comparison with the mitogenomes of *Spodoptera litura* (Fabricius, 1775) (NC_022676), *Agrotis segetum* (Denis & Schiffermüller, 1775) (JX316220), and *Helicoverpa armigera* (Hübner, 1808) (NC_014668). The circular physical maps of the mitogenomes were generated using the Proksee web-based server (Grant et al. 2023) and manually refined with Adobe Illustrator.

### Sequence analysis and codon usage bias

Nucleotide composition, strand asymmetry, and codon usage patterns were analyzed for the complete mitochondrial genomes of the three *Mudaria* species. Strand asymmetry was calculated using the following formulas: AT-skew = (A – T) / (A + T) and GC-skew = (G – C) / (G + C) (Perna and Kocher 1995). To evaluate the codon usage bias, the nucleotide sequences of the 13 PCGs were extracted using a custom Python script. The relative synonymous codon usage (RSCU) was calculated based on the invertebrate mitochondrial genetic code. During this process, leucine (Leu) and serine (Ser) were strictly separated into Leu1/Leu2 and Ser1/Ser2 for independent statistical analysis. The RSCU results were visualized using the ggplot2 package (Wickham 2016) in R (R Core Team 2023).

### Taxon sampling and phylogenetic analysis

Phylogenetic relationships of the genus *Mudaria* within Noctuidae were evaluated based on a mitogenomic super-matrix from 144 species, including three *Mudaria* species (in this study), 138 species in Noctuidae, and three outgroups (belonging to Erebidae, Nolidae, and Notodontidae, respectively) (Suppl. material 1). Considering the limitations of mitochondrial data, we extracted *cox1* gene sequences from more closely related subfamilies flanking the *Mudaria* clade for in-depth analysis (Suppl. material 2).

Nucleotide sequences of the two ribosomal RNAs (*rrnL* and *rrnS*) were aligned individually using MAFFT v. 7 (Katoh and Standley 2013) under the --auto strategy. Nucleotide sequences of the 13 PCGs were aligned based on codon structures using MACSE v. 2.07 (Ranwez et al. 2018) to preserve correct translation reading frames and handle potential frameshifts. Individual alignments were subsequently trimmed using trimAl v. 1.2 (Capella-Gutiérrez et al. 2009) under the -automated1 heuristic mode optimized for maximum-likelihood (ML) analyses. Cleaned single-gene alignments were concatenated into a comprehensive mitogenomic super-matrix using a custom Python script, and frameshift indicators (!) generated by MACSE were converted to standard gaps (-) prior to tree reconstruction.

Phylogenetic trees for both mitogenomic super-matrix and *cox1* were reconstructed under the ML framework using IQ-TREE v. 3.1.0 (Minh et al. 2020). For the 144-species mitogenomic dataset, a partitioned analysis strategy was employed, allowing evolutionary models and substitution parameters to be optimized independently for each gene partition. For the 249-species *cox1* dataset, substitution models were automatically screened under the default ModelFinderPlus (-m mfp) option based on the Bayesian information criterion (BIC), which selected “GTR+F+R7” as the optimal model for the final tree calculation. Node support and branch robustness for both tree topologies were evaluated via 1,000 ultrafast bootstrap replicates (UFBoot2). The resulting tree topologies were visualized and manually formatted for publication.

## Results

### Mitochondrial genome organization and structure

**Figure 2.**
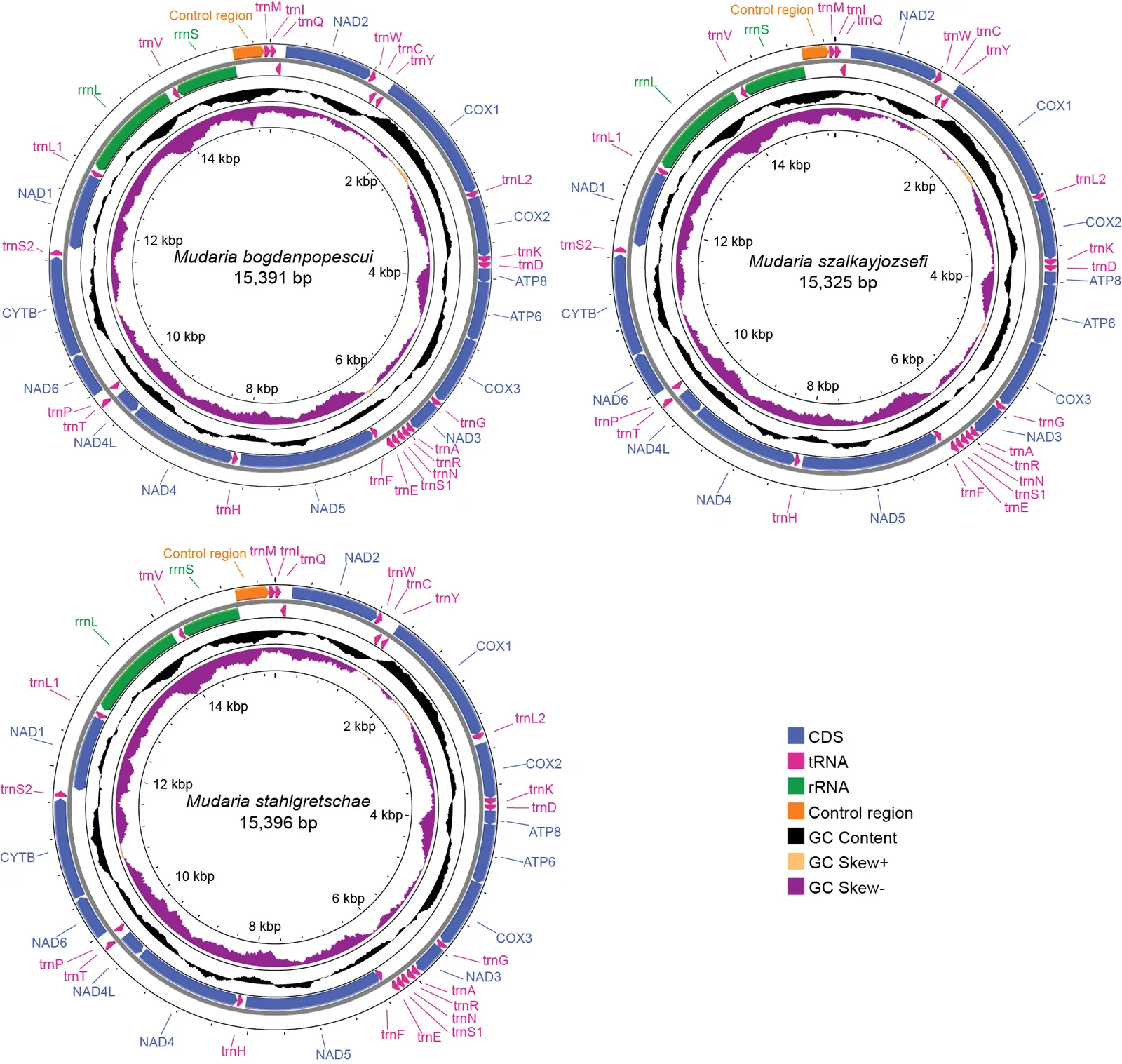
Circular maps representing the complete mitochondrial genome structural architecture of three *Mudaria* species generated via Proksee. The maps illustrate the organization and transcription direction of 37 mitochondrial genes, including 13 protein-coding genes, 22 tRNA genes, and two rRNA genes. The directional rings display the forward and reverse gene transcription layout (outermost), GC content variance (middle), and GC-skew asymmetry parameters (innermost).

The complete mitogenomes of *Mudaria bogdanpopescui*, *M. szalkayjozsefi*, and *M. stahlgretschae* were sequenced and assembled as circular, double-stranded molecules with total lengths of 15,391 bp, 15,325 bp, and 15,396 bp, respectively (Fig. 2; Table 1). All three mitogenomes contain the typical set of 37 genes traditionally conserved in metazoans, comprising 13 protein-coding genes (PCGs), 22 transfer RNA genes (tRNAs), and two ribosomal RNA genes (rRNAs), alongside a non-coding control region (Table 2). The gene arrangement and transcription directions are completely identical among the three species (Fig. 2), perfectly matching the ancestral lepidopteran gene order (Cameron 2014), and no evidence of gene rearrangement, duplication, or inversion phenomena was observed. Within these mitogenomes, 23 genes are encoded on the majority strand (J-strand), while the remaining 14 genes (*nad1*, *nad4*, *nad4l*, *nad5*, eight tRNAs, and two rRNAs) are located on the minority strand (N-strand).

**Table 1.** Nucleotide composition and skewness properties of different mitogenomic regions among *Mudaria bogdanpopescui*, *Mudaria szalkayjozsefi*, and *Mudaria stahlgretschae*.

**Region**	**Species**	**Size (bp)**	**A%**	**T%**	**C%**	**G%**	**A+T%**	**AT-skew**	**GC-skew**
Whole genome	*M. bogdanpopescui*	15,391	39.6	40.9	11.8	7.7	80.5	–0.017	–0.211
	*M. szalkayjozsefi*	15,325	39.4	41.4	11.5	7.6	80.9	–0.025	–0.203
	*M. stahlgretschae*	15,396	39.5	40.6	12.1	7.8	80.1	–0.014	–0.213
PCGs	*M. bogdanpopescui*	11,156	33.1	45.6	10.3	11.0	78.7	–0.158	0.030
	*M. szalkayjozsefi*	11,143	33.2	46.0	10.0	10.8	79.2	–0.161	0.042
	*M. stahlgretschae*	11,140	33.1	45.1	10.7	11.2	78.2	–0.154	0.021
tRNAs	*M. bogdanpopescui*	1,468	41.8	39.8	7.6	10.9	81.5	0.024	0.181
	*M. szalkayjozsefi*	1,461	42.4	39.5	7.6	10.5	81.9	0.036	0.159
	*M. stahlgretschae*	1,467	41.7	39.9	7.8	10.6	81.6	0.023	0.156
rRNAs	*M. bogdanpopescui*	2,042	43.2	41.5	5.0	10.2	84.8	0.020	0.338
	*M. szalkayjozsefi*	2,036	43.4	41.4	5.0	10.2	84.8	0.023	0.342
	*M. stahlgretschae*	1,998	42.9	41.3	5.2	10.6	84.2	0.018	0.346
Control region	*M. bogdanpopescui*	358	45.3	48.3	5.3	1.1	93.6	–0.033	–0.652
	*M. szalkayjozsefi*	308	45.5	48.7	4.9	1.0	94.2	–0.034	–0.667
	*M. stahlgretschae*	385	45.7	47.8	4.2	2.3	93.5	–0.022	–0.280

**Table 2.** Annotation, gene boundaries, and codon parameters of the complete mitogenomes of three *Mudaria* species.

**Gene**	**Strand**	***M. bogdanpopescui***			***M. szalkayjozsefi***			***M. stahlgretschae***		
		**Size (bp)**	**IGN (bp)**	**Codon**	**Size (bp)**	**IGN (bp)**	**Codon**	**Size (bp)**	**IGN (bp)**	**Codon**
*trnI*	+	65	0	—	64	0	—	67	0	—
*trnQ*	–	69	–3	—	69	–3	—	69	–3	—
*nad2*	+	1011	47	ATT/TAA	1011	48	ATT/TAA	1011	60	ATT/TAA
*trnW*	+	66	3	—	67	3	—	67	3	—
*trnC*	–	68	31	—	66	35	—	68	22	—
*trnY*	–	67	34	—	65	29	—	64	34	—
*cox1*	+	1539	2	CGA/TAA	1539	2	CGA/TAA	1539	2	CGA/TAA
*trnL2*	+	67	–5	—	67	–5	—	67	–5	—
*cox2*	+	685	0	ATG/T--	685	0	ATG/T--	669	48	ATG/T--
*trnK*	+	71	–3	—	71	–3	—	71	–3	—
*trnD*	+	67	0	—	67	–1	—	65	4	—
*atp8*	+	165	0	ATC/TAA	159	9	ATA/TAA	165	0	ATA/TAA
*atp6*	+	678	–7	ATG/TAA	678	–7	ATG/TAA	678	–7	ATG/TAA
*cox3*	+	789	–1	ATG/TAA	789	–1	ATG/TAA	789	–1	ATG/TAA
*trnG*	+	65	8	—	65	9	—	65	5	—
*nad3*	+	354	0	ATT/TAA	354	0	ATT/TAA	354	0	ATT/TAA
*trnA*	+	66	1	—	66	2	—	66	3	—
*trnR*	+	65	4	—	64	4	—	65	4	—
*trnN*	+	65	5	—	65	4	—	66	18	—
*trnS1*	+	66	9	—	66	7	—	66	0	—
*trnE*	+	68	5	—	66	3	—	66	1	—
*trnF*	–	67	4	—	67	3	—	67	4	—
*nad5*	–	1716	–1	ATT/TAA	1716	3	ATT/TAA	1719	–17	ATT/TAA
*trnH*	–	67	30	—	67	30	—	67	39	—
*nad4*	–	1336	3	ATG/T--	1336	3	ATG/T--	1336	3	ATG/T--
*nad4l*	–	291	15	ATG/TAA	291	15	ATG/TAA	291	15	ATG/TAA
*trnT*	+	67	4	—	67	7	—	67	6	—
*trnP*	–	65	0	—	65	0	—	66	0	—
*nad6*	+	504	32	ATT/TAA	498	38	ATT/TAA	498	38	ATT/TAA
*cob*	+	1149	8	ATG/TAA	1149	7	ATG/TAA	1152	8	ATG/TAA
*trnS2*	+	65	15	—	67	20	—	65	19	—
*nad1*	–	939	19	ATG/TAA	939	19	ATG/TAA	939	18	ATG/TAA
*trnL1*	–	68	1	—	68	1	—	69	1	—
*rrnL*	–	1262	40	—	1256	29	—	1264	54	—
*trnV*	–	66	67	—	64	67	—	66	65	—
*rrnS*	–	780	0	—	780	0	—	734	0	—
*trnM*	+	68	358	—	68	308	—	68	385	—

### Nucleotide composition and skewness

Nucleotide composition analysis revealed that all three *Mudaria*mitogenomes exhibit a profound bias toward A and T nucleotides, a signature characteristic of ditrysian Lepidoptera (Perna and Kocher 1995). The overall A+T contents of the whole genomes are 80.5% for *M. bogdanpopescui*, 80.9% for *M. szalkayjozsefi*, and 80.1% for *M. stahlgretschae* (Table 1). Among the different functional genomic regions, the control region possesses the highest localized A+T content (93.6%–94.2%), whereas the PCGs exhibit the lowest relative A+T proportions (78.2%–79.2%).

The nucleotide composition asymmetry was measured via skewness metrics. All three species consistently display negative AT-skews (ranging from –0.014 to –0.025) and negative GC-skews (ranging from –0.203 to –0.213) across their entire mitogenomes (Table 1). This explicitly demonstrates that the occurrence of thymine (T) is slightly higher than adenine (A), and the occurrence of cytosine (C) is substantially higher than guanine (G) on the majority strand, revealing a conserved pattern of compositional asymmetry across the genus (Perna and Kocher 1995; Liu et al. 2022).

### Protein-coding genes and relative synonymous codon usage

The total combined length of the 13 PCGs is highly uniform across the three species, measuring 11,156 bp in *M. bogdanpopescui*, 11,143 bp in *M. szalkayjozsefi*, and 11,140 bp in *M. stahlgretschae*. Most PCGs initiate transcription with standard ATN start codons (such as ATG, ATT, ATA, and ATC) and terminate with canonical TAA or TAG stop codons.

However, fine-scale comparative analysis highlighted several key specific features and interspecific variations (Table 2). The *cox1* gene in all three species consistently utilizes the non-canonical tetranucleotide-like start codon CGA, which serves as a widely recognized molecular synapomorphy for macrolepidopteran lineages (Liu et al. 2022). Its transcription terminates cleanly with a standard TAA codon. The *atp8* gene represents a distinct point of sequence divergence among the species; it initiates with an ATC codon in *M. bogdanpopescui*, but switches to an ATA codon in both *M. szalkayjozsefi* and *M. stahlgretschae*. Additionally, while most PCGs possess regular complete trimers at their 3' ends, the *cox2* and *nad4* genes across all three species terminate prematurely with an incomplete single-thymine stop codon (T--). Such incomplete stop codons are structurally rescued via post-transcriptional polyadenylation during mRNA maturation to form functional TAA signals.

**Figure 3.**
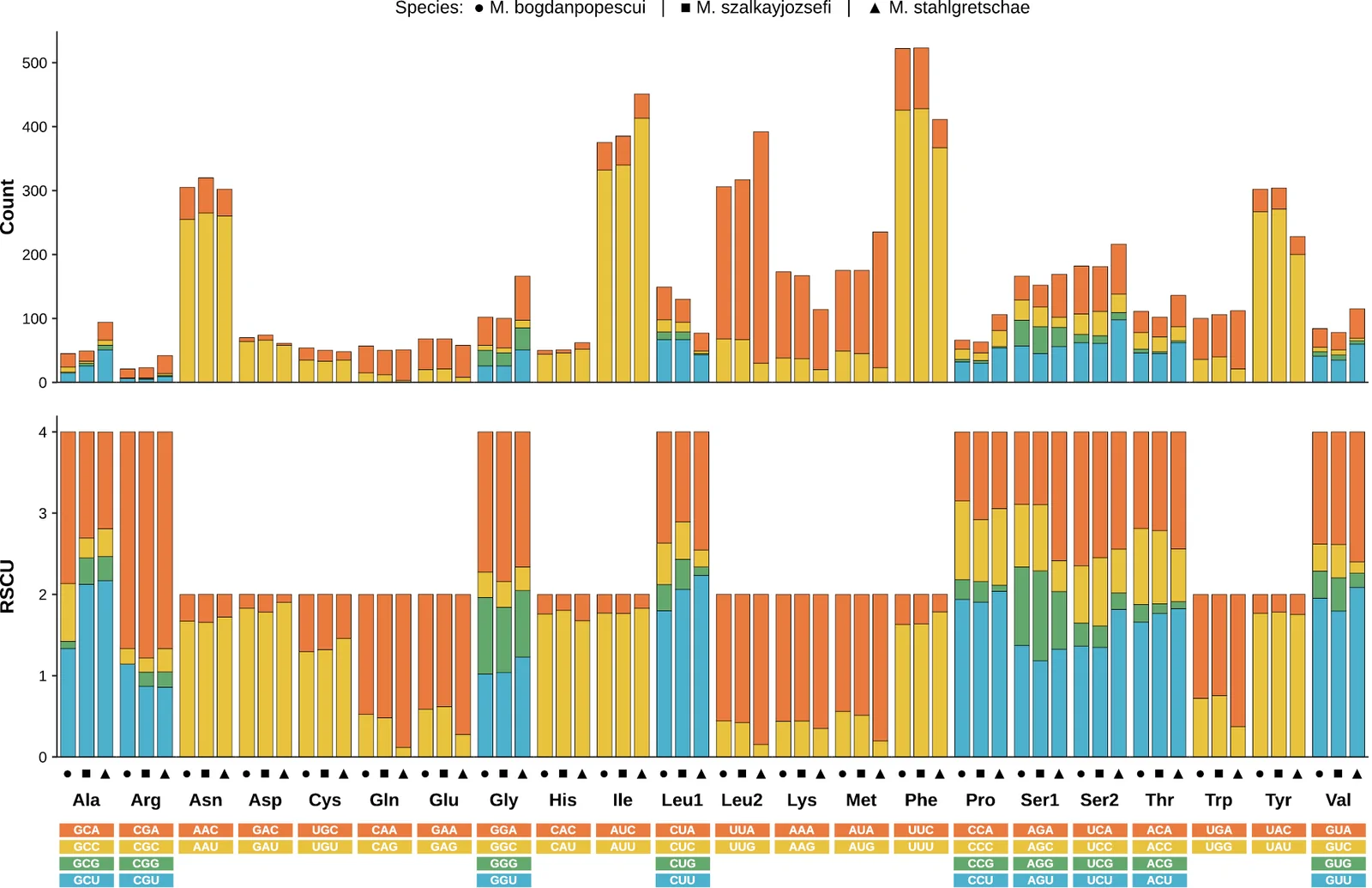
Relative synonymous codon usage (RSCU) of the 13 protein-coding genes in the mitogenomes of the three *Mudaria* species. The x-axis indicates the amino acids and their corresponding synonymous codons, while the y-axis represents the specific RSCU values. The distinct preference for A/U-ending codons reflects the strong A+T mutational bias typical of lepidopteran mitogenomes.

The relative synonymous codon usage (RSCU) values for the 13 PCGs of the three *Mudaria* species were calculated and visualized to evaluate codon usage bias (Fig. 3). The codon usage profiles exhibit a highly conserved and identical pattern across the three species. The most frequently encoded amino acids are Leucine (*Leu*), Isoleucine (*Ile*), and Phenylalanine (*Phe*). Consistent with the profound A+T bias of the overall mitogenomes, there is a strong preference for synonymous codons ending in A or U at the third codon position. Specifically, UUA (*Leu2*), AUU (*Ile*), and UUU (*Phe*) emerged as the most abundantly utilized codons, reflecting a classic mutational pressure toward A/U-rich architectures typical of lepidopteran mitochondrial genomes.

### Transfer and ribosomal RNA genes

In the mitogenomes of the three *Mudaria* species, all 22 typical transfer RNA (tRNA) genes were successfully annotated. They are interspersed throughout the circular genomes, with individual lengths ranging from 64 to 71 bp. The total length of the 22 tRNAs is 1,468 bp in *M. bogdanpopescui*, 1,461 bp in *M. szalkayjozsefi*, and 1,467 bp in *M. stahlgretschae*. The two ribosomal RNA genes, *rrnL* (16S rRNA) and *rrnS* (12S rRNA), are consistently located between *trnL1* and the control region, separated by *trnV*. The combined lengths of the rRNA genes are 2,042 bp, 2,036 bp, and 1,998 bp for *M. bogdanpopescui*, *M. szalkayjozsefi*, and *M. stahlgretschae*, respectively. All rRNA regions exhibit high A+T contents (84.2%–84.8%), which is characteristic of the superfamily Noctuoidea.

### Control region

The control region, representing the largest non-coding AT-rich sequence in the mitogenome, is situated between *rrnS* and *trnM*. Its length is highly variable among the three species, measuring 358 bp in *M. bogdanpopescui*, 308 bp in *M. szalkayjozsefi*, and 385 bp in *M. stahlgretschae*. Notably, this region harbors the most extreme compositional bias within the entire genome, with the A+T content reaching 93.6%, 94.2%, and 93.5%, respectively. This extraordinary A+T richness is typical for its biological function in regulating the initiation of replication and transcription in insect mitochondria.

### Phylogenetic relationships based on mitochondrial and *cox1* datasets

**Figure 4.**
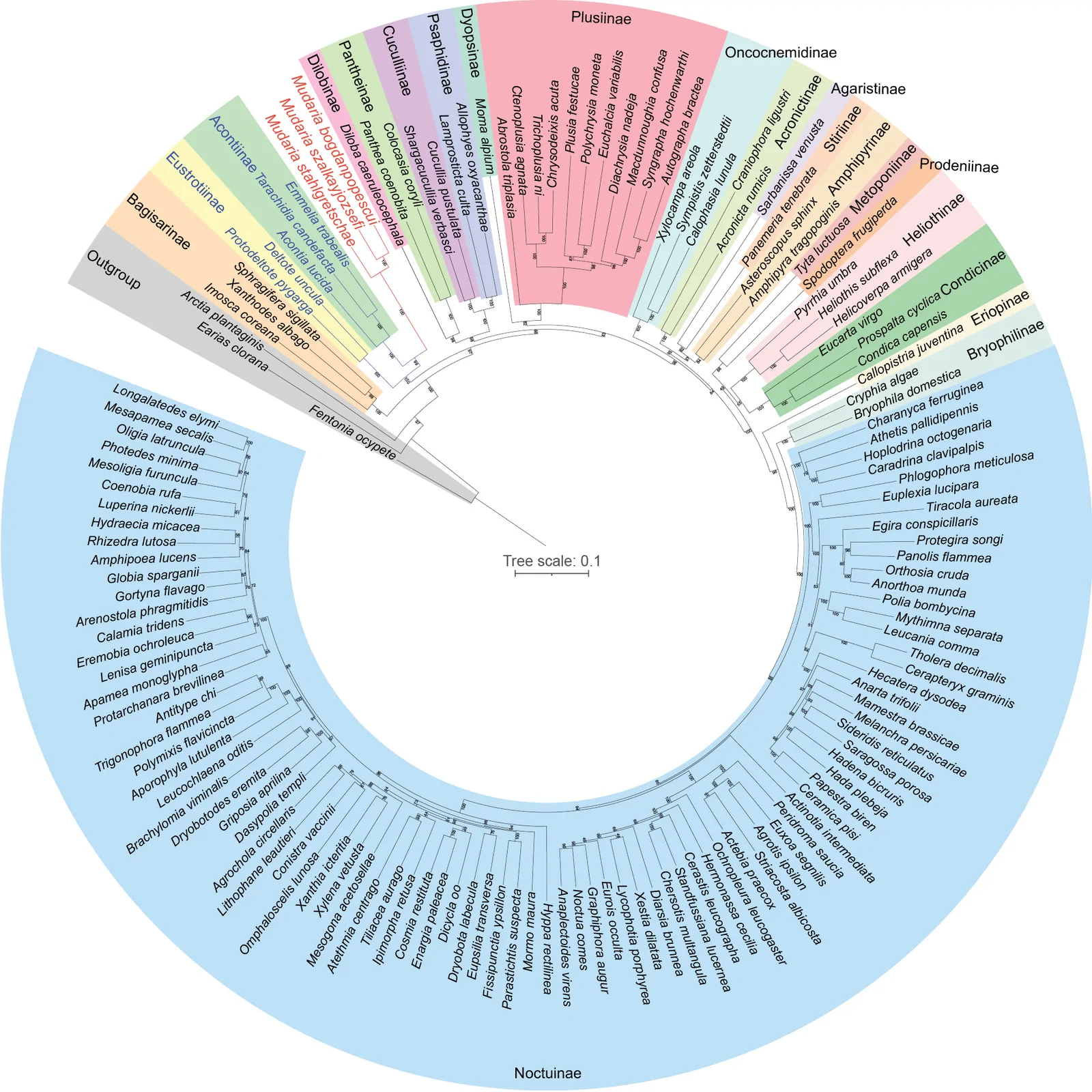
Maximum-likelihood (ML) phylogenetic tree of Noctuidae based on the concatenated mitochondrial super-matrix (13 PCGs + 2 rRNAs) of 144 species. Bootstrap support values are indicated at the respective nodes. The genus *Mudaria* is highlighted, illustrating its distinct phylogenetic placement as the sister group to the subfamily Acontiinae.

Phylogenetic reconstruction based on the concatenated mitochondrial dataset of 144 species successfully resolved the subfamilial backbone of Noctuidae with robust nodal support. The genus *Mudaria* was recovered as a strongly supported monophyletic clade (bootstrap support, BS = 100%). Intriguingly, instead of nesting within the subfamily Noctuinae or Psaphidinae where it has been previously classified (Pellinen et al. 2018; Ronkay et al. 2022, 2023), *Mudaria* was recovered as an independent lineage among the basal subfamilies of Noctuidae, positioned as the sister group to the subfamily Acontiinae (Fig. 4). This high-level topology provides strong molecular evidence suggesting a significant evolutionary divergence of *Mudaria* from the typical Noctuinae lineages.

**Figure 5.**
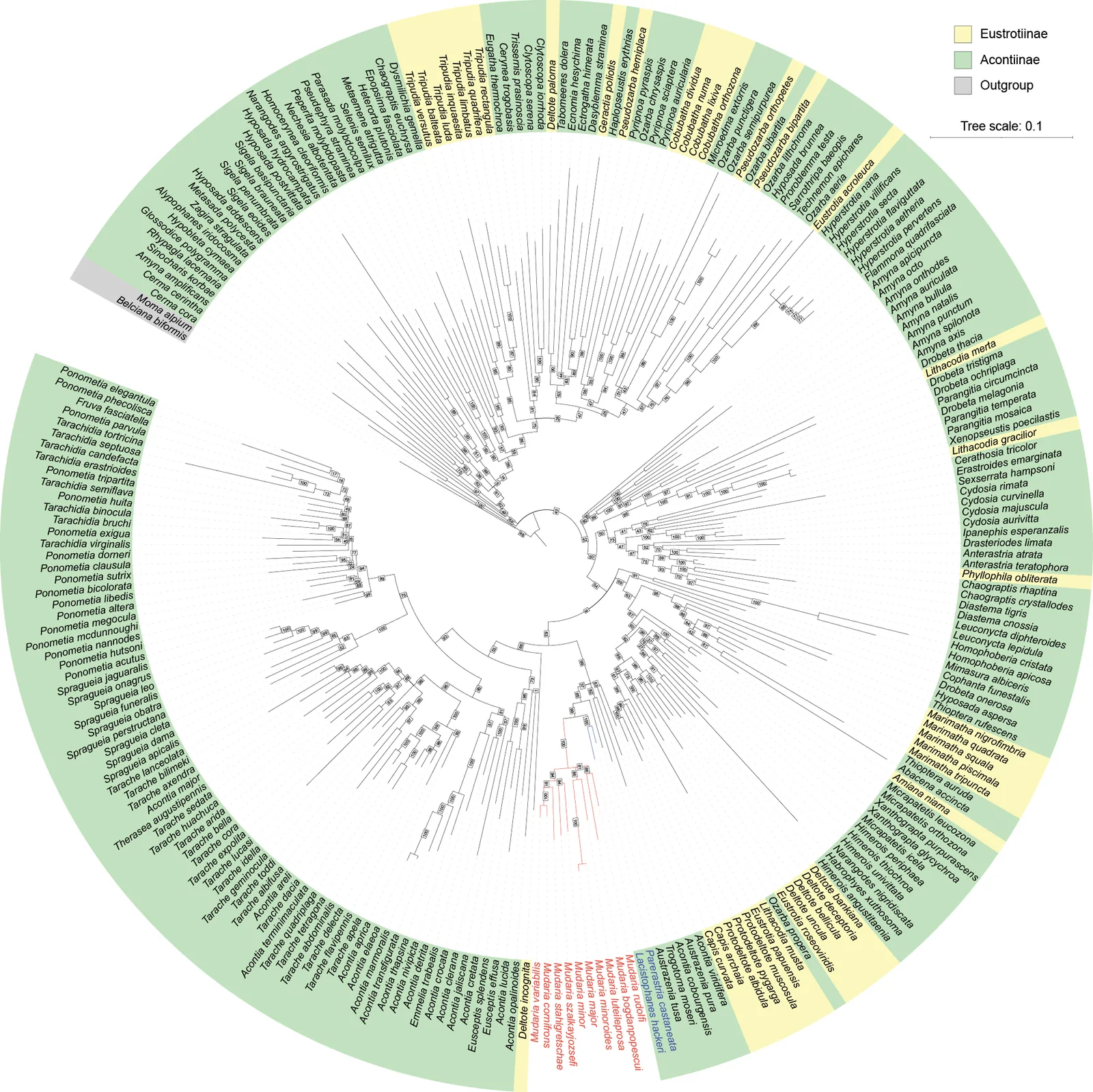
Maximum-likelihood (ML) phylogenetic tree inferred from the expanded *cox1* dataset of 249 species. Bootstrap support values are displayed at the nodes. The tree highlights the clustering of the sampled *Mudaria* species and its sister-group relationship with the lineage comprising *Parerastria castaneata* and *Lacistophanes hackeri*.

To achieve higher taxonomic resolution and clarify localized evolutionary affinities, the expanded 249-species *cox1* barcode dataset was analyzed. All sampled *Mudaria* species, including the three investigated species, clustered together with premium nodal support (BS = 100%) (Fig. 5). Crucially, the entire *Mudaria* clade was resolved as the sister group to a well-supported lineage comprising *Parerastria castaneata* Warren, 1914 and *Lacistophanes hackeri* Turner, 1947. This finding not only corroborates the mitogenomic results but also pinpoints the precise evolutionary neighborhood of *Mudaria*, definitively embedding its generic position in close relation to these specific acontiine lineages.

### Taxonomic considerations

Our molecular data provide preliminary insights into the systematic placement of *Mudaria*. While the current results suggest a close relationship with members of Acontiinae or Eustrotiinae, we note that such a repositioning lacks corroboration from morphological evidence at this stage. Given the absence of detailed comparative morphological data (e.g. male genitalia structures), the definitive subfamilial classification of *Mudaria* remains tentative. Further integrated analyses combining dense molecular sampling and comprehensive morphological descriptions are required to formally establish its taxonomic status.

## Discussion

### Conservation of mitogenome architecture

The newly sequenced complete mitogenomes of *Mudaria bogdanpopescui*, *M. szalkayjozsefi*, and *M. stahlgretschae* exhibit features that are highly conserved and characteristic of the family Noctuidae. All three mitogenomes retain the hypothesized ancestral lepidopteran mitochondrial gene arrangement without any significant gene rearrangements or duplications. The strong A+T bias, negative AT-skew, and the distinct preference for A/U-ending codons revealed by the RSCU analysis align closely with previously documented evolutionary patterns in lepidopteran mitogenomes (Cameron 2014; Liu et al. 2022). Furthermore, the utilization of the non-canonical start codon CGA in the *cox1* gene, a widespread synapomorphy across macrolepidoptera, further confirms the structural and functional stability of the mitochondrial genome within this genus.

### Phylogenetic affinities of *Mudaria* within Noctuidae

The subfamilial classification of the genus *Mudaria* has historically been one of the most contentious topics in noctuid systematics. Over the past few decades, morphological ambiguities have led various authors to assign the genus to widely disparate subfamilies, including Hadeninae (Holloway 1989), Condicinae (Kononenko 2013), Noctuinae incertae sedis (Pellinen et al. 2018), and Psaphidinae (Ronkay et al. 2022, 2023). This remarkable taxonomic instability underscores the limitations of relying solely on homoplasious morphological characters for endophytic fruit-boring lineages, where specialized life histories can severely obscure evolutionary origins.

Our molecular data derived from two robust phylogenetic datasets provide a clear resolution to this long-standing debate by firmly establishing that *Mudaria* represents a basal lineage within Noctuidae, entirely distinct from the derived typical Noctuinae. In the 144-species mitogenomic super-matrix tree, *Mudaria* was recovered as a strongly supported monophyletic clade positioned as the sister group to the subfamily Acontiinae, representing an independent lineage among the basal subfamilies.

To further dissect this localized relationship at a finer taxonomic scale, the expanded 249-species *cox1* dataset was analyzed. The resulting fine-scale topology not only robustly maintained the clustering of the sampled species but also pinpointed their exact evolutionary neighborhood. Specifically, *Mudaria* was resolved as the immediate sister group to the lineage comprising *Parerastria castaneata* and *Lacistophanes hackeri*. This congruent positioning across both datasets demonstrates that the evolutionary trajectory of *Mudaria* is deeply embedded within the basal nocturnal lineages closely associated with the Acontiinae and Eustrotiinae clades rather than typical trifine noctuids.

### Incongruence between deep and local topologies

An interesting aspect of our dual-dataset analysis is the minor topological discrepancy regarding the precise subfamilial placement of *Mudaria*. While the multi-gene mitogenomic tree positions the genus as a distinct sister branch to Acontiinae, the single-gene *cox1* barcode tree embeds it directly adjacent to specific entries traditionally classified under Acontiinae.

Such systematic fluctuations are highly characteristic of the basal lineages of Noctuidae. As comprehensively demonstrated by major modern noctuoid re-evaluations (Zahiri et al. 2011, 2013; Keegan et al. 2021), the deep phylogenetic relationships among basal clades like Acontiinae and Eustrotiinae are notoriously fluid, with subfamilial concepts and boundaries undergoing continuous historical redefinition. The single-gene *cox1* locus, while invaluable for species-level identification and genus-level monophyly validation, frequently suffers from signal saturation or localized lineage sorting when resolving deeper, ancient divergences (Zahiri et al. 2013). Therefore, the distinct sister-group position recovered by the concatenated mitogenomic super-matrix represents a more structurally reliable evolutionary signal, suggesting that *Mudaria* may belong to an autonomous, specialized lineage that split early during the radiation of the basal noctuid groups.

Furthermore, these results yield a compelling methodological insight that serves as a valuable reference for resolving other taxonomically unstable taxa within the hyper-diverse superfamily Noctuoidea. Historically, macro-scale phylogenetic studies have frequently relied on the sparse sampling of “representative” or “typical” species to infer higher-level systematic relationships. Our findings demonstrate that such an approach possesses inherent flaws and significant limitations, as it often fails to capture the true evolutionary positions of atypical or highly specialized lineages like *Mudaria*. Instead, the most robust and reliable strategy requires a “bottom-up” approach: delving deeply into small-scale local groups point-by-point, sequentially connecting these discrete points into lineage-specific lines, and ultimately integrating them into comprehensive phylogenetic surfaces. Although this exhaustive, dense-sampling strategy is undeniably intensive in terms of time, labor, and sequencing resources, it remains the most secure and definitive pathway to achieving a stable classification in such a massively diverse and phylogenetically complex landscape.

### Taxonomic restraint and implications for agricultural biosecurity

Although our molecular phylogenies provide clear, robust coordinates for the evolutionary position of *Mudaria*, we deliberately exercise taxonomic restraint and refrain from publishing a formal subfamilial reassignment or erecting a new nomenclatural status in this study. In traditional lepidopteran taxonomy, a permanent systematic transition requires comprehensive integrative validation, combining mitogenomic data with formal comparative morphology of the adult genitalia, tympanal organs, and larval chaetotaxy, which are currently unavailable for the entire breadth of this genus. Consequently, we tentatively treat *Mudaria* as a distinct lineage closely allied with the basal Acontiinae branch, pending future detailed anatomical dissections. Furthermore, since the mitochondrial genome of the type species, *Mudaria cornifrons*, is currently unavailable and could not be included in our mitogenomic super-matrix, future studies incorporating the mitogenome of the type species will be crucial for definitively resolving the subfamilial assignment.

Beyond its systematic importance, the establishment of this baseline genomic framework has profound practical applications for international trade and agricultural quarantine. Pests within this genus, most notably the durian-fruit borer *M. luteileprosa*, cause catastrophic post-harvest economic losses and trigger strict phytosanitary rejections globally (Jittamai et al. 2024). Because these moths spend most of their destructive larval stages hidden deep inside the pulp of high-value tropical fruits, morphology-based intercept identification at border control points is extremely difficult and error-prone. This diagnostic challenge is further heightened by the co-occurrence of other congeners, such as *M. cornifrons* and *M. variabilis*, which are morphologically nearly indistinguishable from primary pests like *M. luteileprosa* and *M. stahlgretschae*, yet are typically associated with alternative Malvaceae hosts or occupy different seasonal phenological windows (Chamsuk et al. 2026). The complete mitogenomes and curated *cox1* references presented in this study provide highly accurate, rapid molecular diagnosis anchors. This digital genetic resource enables the precise tracking of cryptic larval infestations, bridging basic evolutionary coordinates with applied biosecurity by helping quarantine officers differentiate economic pests from non-target wild species at checkpoints. Ultimately, this genomic framework supports the development of early warning molecular assays and robust integrated pest management (IPM) strategies to protect tropical fruit industries and market access across Southeast Asia.

## Additional information

### Artificial Intelligence (AI) use

The authors accept full responsibility for the content of the manuscript, including the disclosure of any use of AI.

No AI tools were used in the preparation of this manuscript.

### Author ORCIDs

Y. Wang https://orcid.org/0009-0002-3416-1681

Z. Dong https://orcid.org/0000-0002-5040-7904

Z. Deng https://orcid.org/0000-0002-1668-6110

J. He https://orcid.org/0000-0003-1681-9769

L. Zhang https://orcid.org/0000-0001-8042-1419

P. Poolprasert https://orcid.org/0000-0003-3136-0407

H. Han https://orcid.org/0000-0002-2045-6182

X. Li https://orcid.org/0000-0003-0457-7846

### Data availability

All mitochondrial genome sequences produced in this study have been deposited in the GenBank database under the accession numbers PZ387140 (Mudaria bogdanpopescui), PZ387122 (Mudaria szalkayjozsefi) and PZ459543 (Mudaria stahlgretschae).

## Supplementary materials

Supplementary material 1 List of 144 species utilized in the mitogenomic phylogenetic analysis Data type xlsx https://binary.pensoft.net/file/1829782This dataset is made available under the Open Database License (http://opendatacommons.org/licenses/odbl/1.0/). The Open Database License (ODbL) is a license agreement intended to allow users to freely share, modify, and use this Dataset while maintaining this same freedom for others, provided that the original source and author(s) are credited. Yangjie Wang, Zhiwei Dong, Zonghan Deng, Jinwu He, Yuanrong Li, Lijie Zhang, Pisit Poolprasert, Markku Pellinen, Huilin Han, Xueyan Li

Supplementary material 2 List of 249 species utilized in the *cox1*-based fine-scale phylogenetic analysis Data type xlsx https://binary.pensoft.net/file/1829783This dataset is made available under the Open Database License (http://opendatacommons.org/licenses/odbl/1.0/). The Open Database License (ODbL) is a license agreement intended to allow users to freely share, modify, and use this Dataset while maintaining this same freedom for others, provided that the original source and author(s) are credited. Yangjie Wang, Zhiwei Dong, Zonghan Deng, Jinwu He, Yuanrong Li, Lijie Zhang, Pisit Poolprasert, Markku Pellinen, Huilin Han, Xueyan Li
